# The disease burden of multimorbidity and its interaction with educational level

**DOI:** 10.1371/journal.pone.0243275

**Published:** 2020-12-03

**Authors:** Yi Hsuan Chen, Milad Karimi, Maureen P. M. H. Rutten-van Mölken

**Affiliations:** 1 Erasmus School of Health Policy and Management, Erasmus University Rotterdam, Rotterdam, The Netherlands; 2 Institute for Medical Technology Assessment, Erasmus University Rotterdam, Rotterdam, The Netherlands; Osakidetza Basque Health Service, SPAIN

## Abstract

**Introduction:**

Policies to adequately respond to the rise in multimorbidity have top-priority. To understand the actual burden of multimorbidity, this study aimed to: 1) estimate the trend in prevalence of multimorbidity in the Netherlands, 2) study the association between multimorbidity and physical and mental health outcomes and healthcare cost, and 3) investigate how the association between multimorbidity and health outcomes interacts with socio-economic status (SES).

**Methods:**

Prevalence estimates were obtained from a nationally representative pharmacy database over 2007–2016. Impact on costs was estimated in a fixed effect regression model on claims data over 2009–2015. Data on physical and mental health and SES were obtained from the National Health Survey in 2017, in which the Katz-10 was used to measure limitations in activities of daily living (ADL) and the Mental Health Inventory (MHI) to measure mental health. SES was approximated by the level of education. Generalized linear models (2-part models for ADL) were used to analyze the health data. In all models an indicator variable for the presence or absence of multimorbidity was included or a categorical variable for the number of chronic conditions. Interactions terms of multimorbidity and educational level were added into the previously mentioned models.

**Results:**

Over the past ten years, there was an increase of 1.6%-point in the percentage of people with multimorbidity. The percentage of people with three or more conditions increased with +2.1%-point. People with multimorbidity had considerably worse physical and mental health outcomes than people without multimorbidity. For the ADL, the impact of multimorbidity was three times greater in the lowest educational level than in the highest educational level. For the MHI, the impact of multimorbidity was two times greater in the lowest than in the highest educational level. Each additional chronic condition was associated with a greater worsening in health outcomes. Similarly, for costs, where there was no evidence of a diminishing impact of additional conditions either. In patients with multimorbidity total healthcare costs were on average €874 higher than in patients with a single morbidity.

**Conclusion:**

The impact of multimorbidity on health and costs seems to be greater in the sicker and lower educated population.

## Introduction

Multimorbidity, defined as two or more chronic conditions occurring in one person at the same time, is an increasing problem, particularly in countries with a rapidly ageing population [1, 2]. The number of patients with multimorbidity in Europe is around 50 million and is increasing every year [2]. Compared to patients without multimorbidity, those with multimorbidity have a higher mortality rate [3, 4], a higher rate of polypharmacy [5], worse health-related quality of life (HRQoL) [6–8], higher healthcare costs [9–11], and more productivity loss [12] after adjustment of age and other confounders. Moreover, they are disproportionately affected by the fragmentation and the single-disease orientation of the health and social care system [13]. The need for investments in potential solutions such as whole-system, person-centred integrated care is widely recognized. To justify and plan such investments and ensure access for all people with multimorbidity across the layers of the population, it is crucial to have accurate data on the (change in) prevalence of multimorbidity and its impact on physical and mental health, and costs.

Prevalence estimates of multimorbidity and their increase with age and over time vary considerably due to differences in definitions of multimorbidity and sources of data used [14–18]. The most up to date prevalence estimate for the Netherlands was 31.8% in 2018 [19], which was based on a general practitioner (GP) registry. An estimate of (change in) prevalence that is based on a national database representing the entire Dutch population is lacking.

Several studies have investigated the association between multimorbidity and health outcomes, both physical [6–8, 20–22], and mental [6, 8, 23, 24], but many were either small [7, 21], not representative for the entire country [8, 23], focused on a specific subgroup like elderly [6, 7, 21, 22], or focused on a limited number of chronic diseases [24–26]. There are many papers on socio-economic inequalities in health, which clearly demonstrate that lower SES is associated with greater mortality [27, 28], higher prevalence of multimorbidity [29] and worse health outcomes [30] To the best of our knowledge, none of them investigated how the association between multimorbidity and health varies by SES.

The impact of multimorbidity on costs varies by healthcare system and methods used to derive costs [10, 31]. In a recent review study, the ratios of multimorbidity costs to non-multimorbidity costs ranged from 2–16 [32]. Most of the studies in this review were cross-sectional and half of them were based on U.S. data. There is no longitudinal study on the impact of multimorbidity on healthcare costs in the Netherlands.

The aim of our study was to fill in the information gaps identified above. This study provides recent estimates of the prevalence of multimorbidity in the Netherlands and its change over time. Furthermore, we investigated the association between multimorbidity and physical health, mental health and healthcare costs, using databases that are representative for the entire country. In addition, we investigated how educational level interacts with the association between multimorbidity and health.

## Methods

We have used data from a nationally representative pharmacy database, claims database and a health survey to estimate the change in prevalence of multimorbidity and the impact of multimorbidity on health and healthcare costs. All databases used in this study were provided by Statistics Netherlands (CBS, Centraal Bureau voor de Statistiek), the national statistics office of the Netherlands. The study population was the entire adult population (18 years old and over) in the Netherlands. The prevalence estimation was based on data from 2006 to 2016, the association between multimorbidity and health outcomes was based on data from 2017, and the estimated impact of multimorbidity on healthcare costs was based on panel data from 2009 to 2015.

### Identification of chronic conditions and prevalence of multimorbidity

To estimate the prevalence, we used a longitudinal pharmacy database that contained data from everyone in the Netherlands that received medication included in the basic health insurance package, which is approximately 11 million adults (85% of total population) each year [33]. In the Netherlands, every resident is mandatorily enrolled in basic health insurance. All prescribed medicines covered by the basic insurance, including those prescribed by GP’s, to hospital outpatients and patients in residential homes, are recorded in this dataset. The medication prescribed during in-patient hospital stays and over-the-counter medicines are not included in the pharmacy database.

The individual-level prescriptions of medications are coded and categorized using the Anatomical Therapeutic Chemical (ATC) classification system, up to the third level, which is the pharmacological subgroup level [34]. We matched these ATC codes to a list of 21 chronic conditions: acid-related disorders, bone diseases, cancer, cardiovascular diseases, dementia, diabetes mellitus, epilepsy, glaucoma, hyperuricemia (gout), chronic viral infection, hyperlipidemia, intestinal inflammatory diseases, iron deficiency anemia, migraines, pain, Parkinson’s disease, psychological disorders, psychoses, respiratory illness, thyroid disorders, and tuberculosis. To do so we used the Swiss classification by Huber et al. [35], which was an updated version of the original Dutch classification by Lamers et al. [36], that was used in the risk equalization scheme of the Dutch health insurance. Hence this classification was deemed suitable to be used in a study addressing the entire adult population in the Netherlands.

In line with the WHO definition of multimorbidity, adults with two or more chronic conditions in the same year were defined as having multimorbidity [37]. To avoid overestimating the prevalence of chronic conditions, we only labeled a condition as chronic when medication for a certain chronic disease was prescribed at least two years in a row. This continuity criterium was used by previous studies [38, 39].

To estimate the prevalence of multimorbidity by gender and age, the pharmacy database was linked to the municipal basic administration (GBA, Gemeentelijke Basis Administratie) database from CBS, which contains demographic information (year of birth and gender) for all residents in the Netherlands [40]. Data were linked by CBS, using a pseudonymized common ID that is unique for each individual. Information on annual size of the total population in the Netherlands was extracted from StatLine [41].

### Health outcomes

To estimate the association between multimorbidity and health outcomes, which include physical and mental health outcomes, we used data from the Health Survey database [42]. This database contains data from a nation-wide, cross-sectional, annual Health Survey on health and the need for healthcare in the Netherlands, including self-reported physical and mental health, and demographic information such as age, gender, and highest education level. Approximately 15,000 participants across the country are sampled for the survey each year, with a response rate of 63% in 2017 (approximately 9,500 respondents), from which we selected all 7,741 respondents that were 18 years and older in the year 2017. The average age of this dataset is 52.8 years old, 50.5% of the respondents are male (S3 Table). The sample is distributed evenly over all months of the year. First, persons are asked to participate via the internet. Non-responders are re-approached to participate in a face-to-face interview by way of Computer-Assisted Personal Interviewing. A correction is applied to control for differences between the sample and the population. For this purpose, a weighting factor is used based on sex, age, migration background, marital status, degree of urbanisation, province, household size, income, wealth, and survey season. The background characteristics of the Health Survey responders were proven by CBS to be similar to the general population in the Netherlands [42].

Physical health was measured with the Katz-10. This is a validated questionnaire [43, 44], which consists of ten questions asking respondents whether they need help with ADL such as eating, dressing, personal hygiene, mobility in getting up from bed, dressing, transferring, and use of the toilet [45]. The five response options ranged from no difficulty to not even with the help of others. Participants under 54 years only had to answer three of these ten questions, i.e., about their ability to get up from a chair, to get up from a bed and walk up a flight of stairs. For both age groups, responses were linearly transformed onto a scale from 0 (best) to 100 (worst) [46].

Mental health was measured by the Mental Health Inventory 5 (MHI-5). This is a validated questionnaire [47], which consists of five questions asking how much of the time during the last four weeks the respondents had felt happy, calm and peaceful, nervous, downhearted and blue, and so down in the dumps that nothing could cheer them up [48]. The six response options ranged from none of the time to all of the time. Responses were linearly transformed onto a scale from 0 (worst) to 100 (best) [49].

The Health Survey database also contains age, gender, and highest education level achieved, which was classified into five levels, i.e. primary education; pre-vocational training; high school or vocational training; higher education until Bachelor; and master/doctorate [50]. The multimorbidity status of the Health Survey respondents was obtained by linking to the prevalence data (2016). In all the databases provided by CBS, each individual has one unique, encrypted RIN code. This RIN code enable us to link several databases on individual level.

### Healthcare costs

To estimate the impact of multimorbidity on healthcare expenditure, we used a database of health insurance data, containing annual individual-level claims data from 2009 to 2015 [51]. Sixteen categories of costs are included, that cover all expenditures reimbursed by the compulsory basic health insurance in the Netherlands, including GP care, hospital care, dental care, pharmaceuticals, other costs (e.g. paramedic, assistive device and birth care), and total costs. Costs of GP care include the annual per capita registration fee, the consultation fees and other costs incurred by the GP. Costs of hospital care include both inpatient and outpatient hospital care. Pharmacy costs include all the costs of medicines, except for the ones administered during a hospital admission. From the health insurance database, we included only the adult population (18 or older), which was around 2,450,000 each year in the period from 2009 to 2015 (17,048,049 observations for adults across these years in total).

As healthcare costs increase substantially in the year of death [52, 53], it was essential to have mortality data to know whether an individual had died in a certain year. Mortality data for all deceased Dutch residents were obtained from the National Mortality Database, which includes the date and cause of death of all residents in the Netherlands. End-of-life costs were defined as the healthcare expenditures during the year of death.

### Statistical analyses

#### Prevalence

For each year from 2007 to 2016, the prevalence of multimorbidity was calculated as the number of adults with multimorbidity (in a certain age class) divided by the number of adults (in that age class) living in the Netherlands that year. We further classified patients according to their number of chronic conditions (0, 1, 2, 3, 4, and 5 or more) and estimated the prevalence of each class.

#### Health outcomes

To analyse the association between multimorbidity and ADL, we estimated two-part models (2PM), because a large proportion of the people has a score of 0, indicating no problems with their ADL [45]. This makes the conventional ordinary least squares (OLS) inappropriate. In the first part of the 2PM, we used a logit model to estimate the probability of having an ADL score higher than 0, including morbidity status (0 for no morbidity, 1 for single morbidity, and 2 for multimorbidity), age, gender and educational level:

$$P_{r}(ADL_{i}>0|X_{i})=\frac{e^{\alpha +\beta \cdot x_{i}}}{1+e^{\alpha +\beta \cdot x_{i}}}$$ (1)

Where *P*_*r*_ (*ADL*_*i*_ > 0|*X*_*i*_) is the probability of ADL score to be positive with given age, gender, multimorbidity status, and education level, *α* is the constant, and *β*_*i*_ are parameters in the model, which are age, gender, multimorbidity status and education level.

The second part of the 2PM estimated the ADL score among those with a non-zero score, using a generalized linear model with a gamma distribution and log-link function, because the data were skewed to the right. The choice of the model was driven by the Manning and Mullahy algorithm [54]. Like in the first part, the model included morbidity status, age, gender and educational level:

$$\text{ln}(E(ADL_{i}))=\;\alpha +\beta \cdot x_{i}+\varepsilon$$ (2)

Where *ε* stands for the error term. The results of the 2PM combined the probability estimated in the first part and the ADL score estimated in the second part:

$$E(ADL_{i}|x_{i})=P_{r}(ADL_{i}>0|X_{i})\cdot E(ADL_{i}|x_{i},\;ADL_{i}>0)$$ (3)

To analyse the association between multimorbidity and MHI score, we estimated a generalized linear model with the same independent variables as above. Based on the Manning and Mullahy algorithm [54], the identity link function and normal distribution were chosen in this analysis.

$$MHI_{i}=\alpha +\beta \cdot x_{i}+\varepsilon$$ (4)

To investigate whether the association between multimorbidity and ADL or MHI scores differed by level of education, interaction variables of morbidity status and level of education were added to the models above.

#### Healthcare costs

We used fixed-effects regression models to estimate the associations between multimorbidity and healthcare costs [55]. A major concern for observational studies involving human behaviours is the presence of unmeasured potential confounding factors. With the panel dataset that provided individual-level information, we addressed this concern by the ‘within estimation’ of a fixed-effect model, which takes each individual as its own control and estimates the change in healthcare costs of each individual after the morbidity status changed and cancels out those unmeasured, time-invariant confounding factors such as medical care-seeking behaviour [55].

In all models the three-level morbidity status (i.e., 0, 1 for single morbidity, 2 for multimorbidity (i.e., 2+)) was replaced by a six-level morbidity status (i.e., 0, 1, 2, 3, 4, and 5 or more) to investigate the impact of the number of morbidities on health outcomes and costs. All analyses were conducted using Stata 14 statistical software.

## Results

### Prevalence

**Fig 1:**
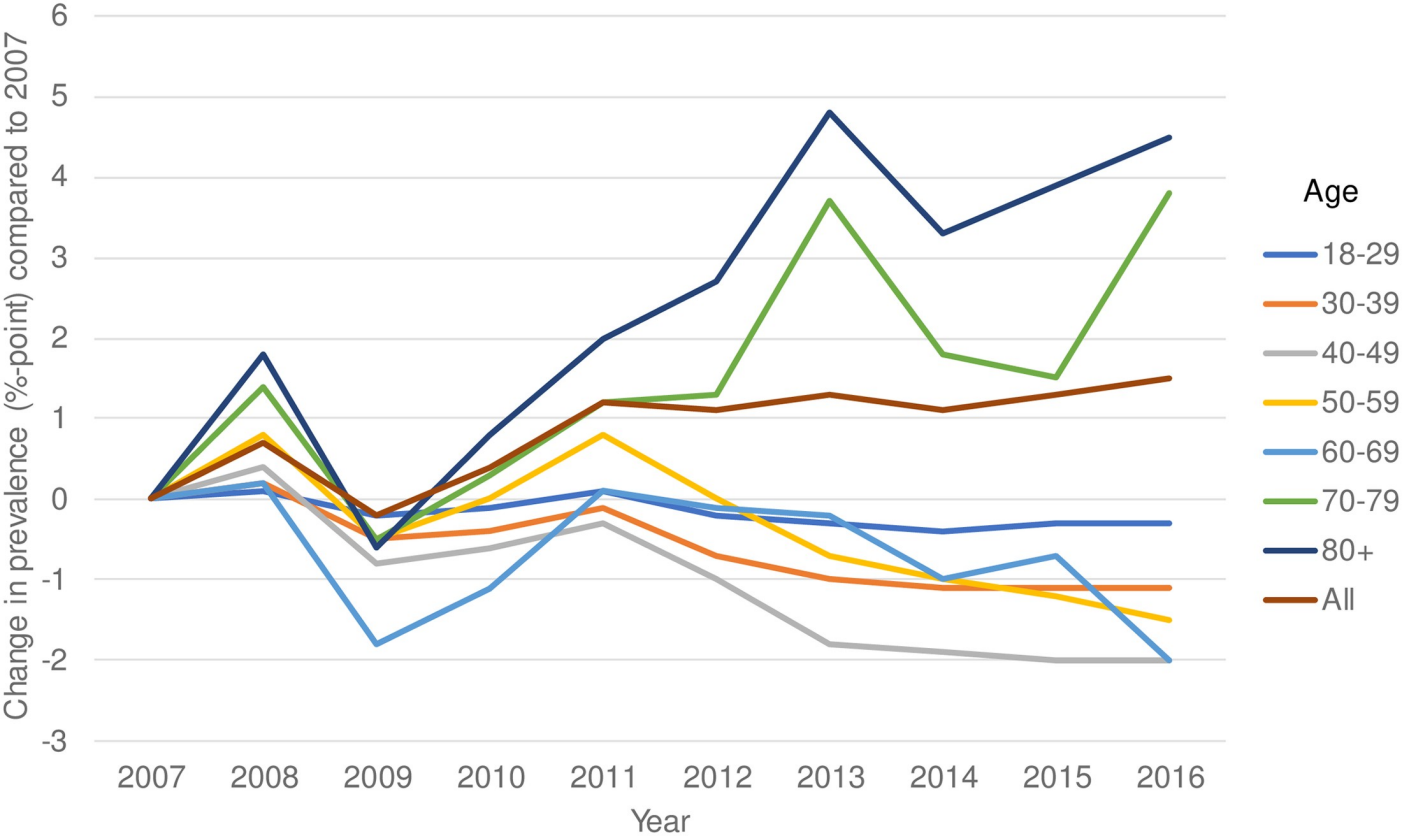
Change in prevalence or multimorbidity by age groups compared to 2007.

**Fig 2:**
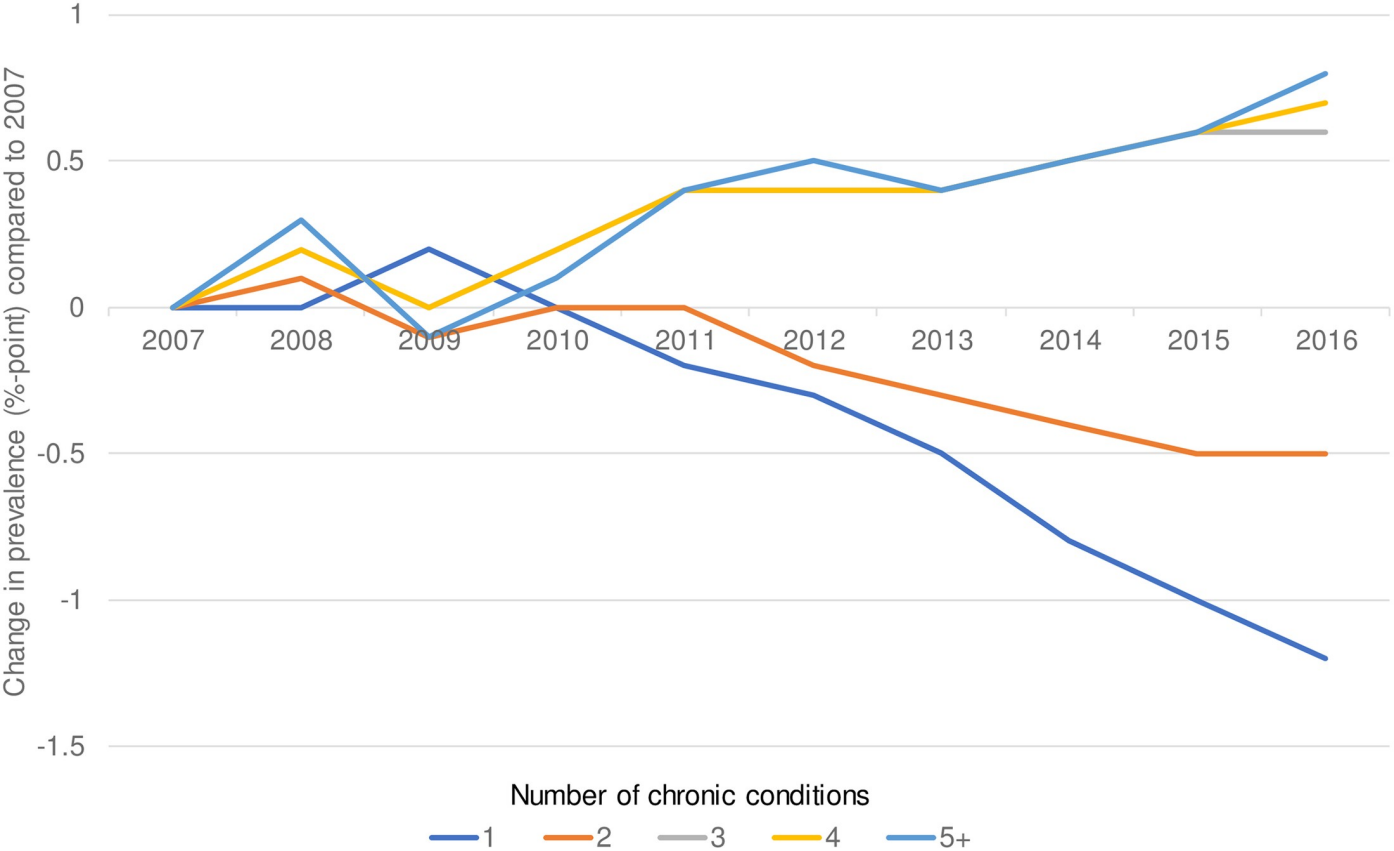
Change in prevalence of different numbers of chronic conditions compared to 2007.

The prevalence of multimorbidity in Dutch adults was 23.2% in 2007 and had increased to 24.7% in 2016 (S1 Table). This raise was primarily due to an increase in those aged over 69 (Fig 1). Compared to 2007, the prevalence has risen with 1.5% points. Over the past ten years, there was a considerable increase in the proportion of people with three or more chronic conditions (Fig 2).

According to the prevalence estimations in 2016, the prevalence of multimorbidity increased sharply with age to 76.2% in the 80 years old or older.

### Health outcomes

Table 1 shows the association between multimorbidity and health outcomes, both for physical and mental health. The odds ratio of having a non-zero ADL score (i.e. having ADL problems) was 3.8 (13.17/3.43) times higher for the multimorbidity group than for the single morbidity group, after adjustment for differences in age, gender, and education level (Table 1). Among those with a non-zero ADL score, the ADL score of those with multimorbidity was 2.2 (1.2*10.6–1.4*10.6) units worse than the ADL score of those with a single morbidity. Combining the results of both parts, the ADL score of the multimorbidity group was 3 units worse than the ADL score of the single morbidity group. Table 1 also shows that, compared to the non-morbidity group, the MHI score worsened by 1.7 units in the single morbidity group and 5 units in the multimorbidity group.

**Table 1:** Associations between multimorbidity and health outcomes.

Health outcome	ADL (Physical health)						MHI (Mental health)		
Model	Part 1: Logistic			Part 2: GLM			OLS		
	OR	95% CI		Exp(b**)	95% CI		b**	95% CI	
Age	1.03	1.02	1.03	0.99	0.99	0.99	0.09	0.08	0.10
Female	1.40	1.23	1.58	1.16	1.06	1.28	-1.69	-2.04	-1.33
Education level* (reference: 1)									
2	0.76	0.62	0.93	0.78	0.67	0.89	0.58	-0.04	1.20
3	0.64	0.52	0.77	0.67	0.59	0.77	1.68	1.11	2.25
4	0.58	0.46	0.73	0.61	0.51	0.72	2.21	1.57	2.85
5	0.39	0.29	0.53	0.70	0.55	0.89	2.09	1.34	2.84
Morbidity condition (reference: no morbidity)									
Single morbidity	3.43	2.67	4.40	1.17	0.93	1.47	-1.72	-2.20	-1.25
Multimorbidity	13.17	10.55	16.44	1.43	1.17	1.76	-5.00	-5.44	-4.55
Constant	0.05	0.04	0.07	10.6	8.56	13.20	5.87	5.82	5.93

The number of participants is 7,741. The part 1 model of ADL is calculated with Eq (1); the part 2 model of ADL is calculated with Eq (2); the model of MHI is calculate with Eq (4).

*Education level: 1 for primary school; 2 for Pre-vocational training; 3 for High school or vocational training; 4 for Higher education until Bachelor; 5 for Master/doctorate.

**b: regression-coefficient

Abbreviations. ADL: activities of daily living, MHI: mental health inventory, OR: odds ratio, GLM: generalized linear model, OLS: ordinary least squares, CI: confidential interval.

**Fig 3:**
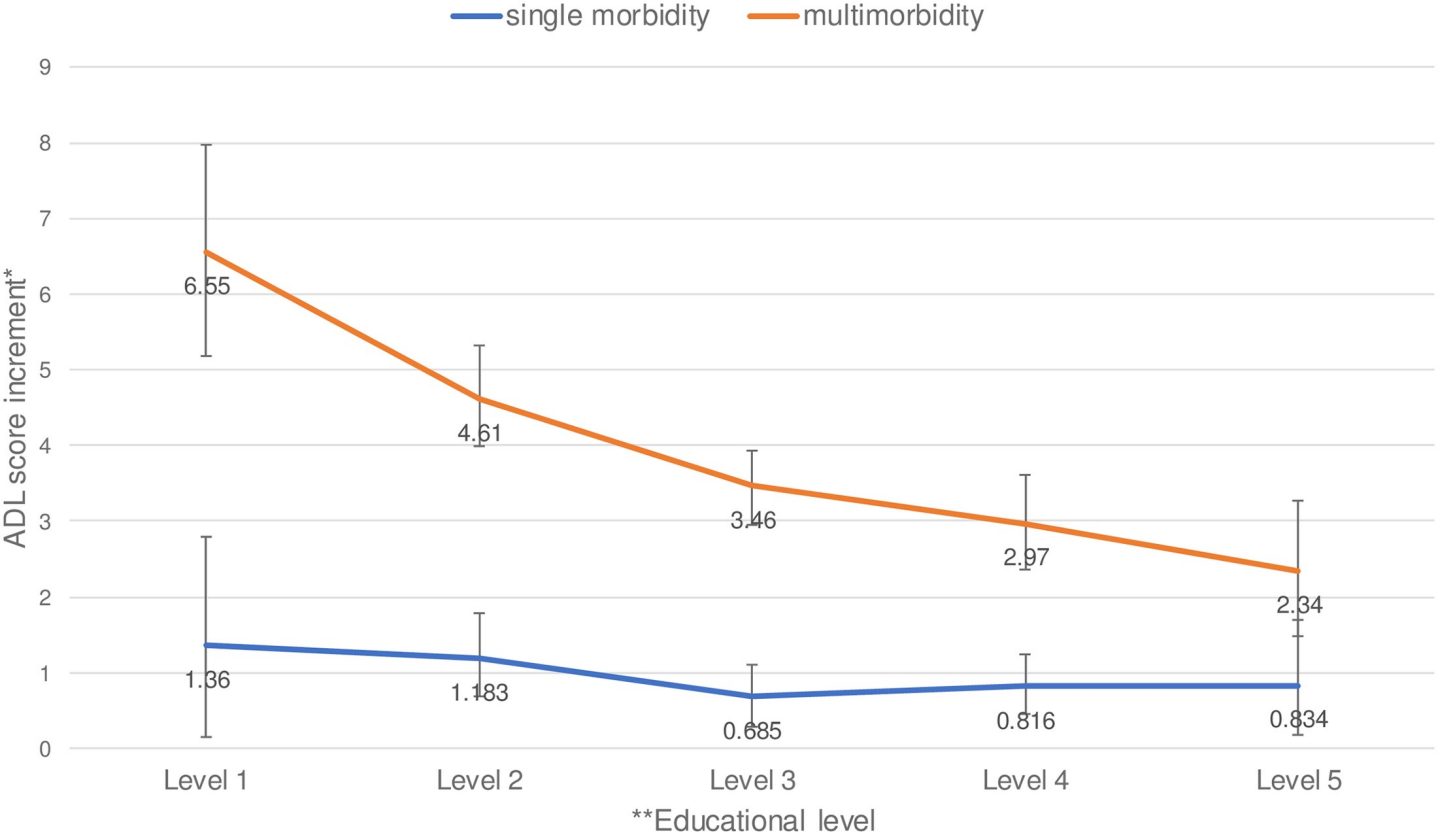
Difference in ADL score compared to no morbidity across education levels. The number of participants is 7,741. The group without a chronic condition is the reference group. *: A higher score indicates a worse ADL. The standard error bars were based on bootstrapping. **Education level: 1 for primary school; 2 for Pre-vocational training; 3 for High school or vocational training; 4 for Higher education until Bachelor; 5 for Master/doctorate.

**Fig 4:**
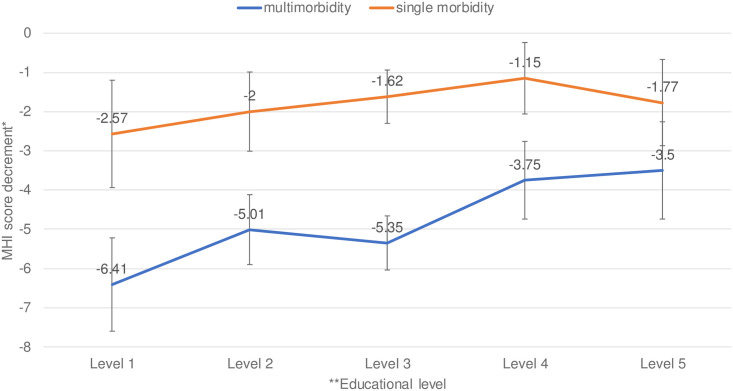
Difference in MHI score compared to no morbidity across education levels. The number of participants is 7,741. The group without a chronic condition is the reference group. *: A lower score indicates a worse mental health. The standard error bars were based on bootstrapping. **Education level: 1 for primary school; 2 for Pre-vocational training; 3 for High school or vocational training; 4 for Higher education until Bachelor; 5 for Master/doctorate.

The regression results showing the association between multimorbidity and physical and mental health outcomes across different education levels are reported in S2 Table. Fig 3 shows the interaction between educational level and the impact of multimorbidity on ADL, and Fig 4 does so for the MHI. The lower the education level, the greater the difference in ADL score and MHI score between multimorbidity and no morbidity. Compared to the non-morbidity group, the ADL score was 6.6 units worse in the group with the lowest educational level, whereas it was only 2.3 units worse in the group with the highest educational level. Compared to the non-morbidity group, the MHI score was 6.4 units worse in the group with the lowest educational level, whereas it was only 3.5 units worse in the group with the highest educational level.

Table 2 shows the association between the number of chronic conditions and the ADL and MHI scores. The group with two chronic conditions had an ADL score that was 1.7 units (calculated with Eq (3)) worse than the group with no chronic conditions, whereas the group with five or more chronic conditions had and ADL score that was 10 units (calculated with Eq (3)) worse. The MHI score for the group with two chronic conditions was 3.4 units worse than that of the group with no chronic conditions, whereas that of the group with five or more chronic conditions was 8.7 units worse.

**Table 2:** Associations between chronic conditions and health outcomes (N = 7,741).

Health outcome	ADL						MHI		
Model	Part 1: Logistic			Part 2: GLM			OLS		
	OR**	95% CI		Exp(b**)	95% CI		b**	95% CI	
Age	1.02	1.02	1.03	0.99	0.98	0.99	0.10	0.09	0.12
Female	1.36	1.19	1.55	1.15	1.05	1.26	-1.59	-1.94	-1.24
Education level* (reference: 1)									
2	0.76	0.61	0.95	0.77	0.67	0.89	0.54	-0.07	1.16
3	0.69	0.56	0.84	0.68	0.59	0.78	1.48	0.92	2.05
4	0.63	0.49	0.80	0.63	0.53	0.75	2.02	1.39	2.66
5	0.42	0.31	0.58	0.68	0.54	0.87	1.88	1.13	2.62
Number of chronic conditions (reference: no chronic condition)									
1	3.53	2.75	4.53	1.18	0.95	1.48	-1.79	-2.26	-1.32
2	6.87	8.80	8.80	1.11	0.89	1.39	-3.41	-3.96	-2.86
3	11.03	14.33	14.33	1.12	0.94	1.47	-4.41	-5.07	-3.75
4	18.35	24.06	24.06	1.40	1.12	1.75	-5.88	-6.61	-5.14
5 or more	47.10	61.97	61.97	1.91	1.55	2.37	-8.65	-9.35	-7.96
Constant	0.05	0.06	0.06	10.54	8.51	13.04	5.89	5.84	5.95

The number of participants is 7,741. The part 1 model of ADL is calculated with Eq (1); the part 2 model of ADL is calculated with Eq (2); the model of MHI is calculate with Eq (4).

*Education level: 1 for primary school; 2 for Pre-vocational training; 3 for High school or vocational training; 4 for Higher education until Bachelor; 5 for Master/doctorate.

**OR: odds ratio, b: regression-coefficient

As the number of chronic conditions increased, the difference in ADL score between two consecutive groups increased as well; i.e., the difference between the group with one chronic condition and the group with two chronic conditions was 0.8 units, whereas the difference between the group with three chronic conditions and the group with four chronic conditions was almost two units (Table 2). The same picture was seen for the MHI.

### Healthcare costs

Table 3 shows the association between multimorbidity and healthcare costs. Costs are presented in Euros (2015 exchange rate: 1$US = 0.92 €). Compared with those in the single-morbidity group, the mean total health costs of the multimorbidity group was € 874 higher. The costs of GP care, pharmaceuticals, and hospital care were € 25, € 226, and € 355 higher in multimorbidity patients than in single-morbidity patients.

**Table 3:** Associations between multimorbidity and healthcare costs (in Euro).

Cost types	Total cost			GP cost			Pharmacy cost			Hospital care cost		
	b**	95% CI		b**	95% CI		b**	95% CI		b**	95% CI	
Morbidity condition (reference: no chronic condition)												
Single morbidity	613.0	607.7	618.4	21.8	21.7	21.9	140.3	139.6	141.1	316.0	312.1	320.0
Multimorbidity	1,487.0	1,479.9	1,494.2	47.4	47.2	47.5	365.9	364.9	366.9	661.3	656.0	666.6
Death*	3,685.0	3,670.3	3,699.7	199.0	198.8	199.3	-266.8	-269.0	-264.7	2,998.0	2,987.2	3,008.9
Year (reference: 2009)												
2010	119.0	115.0	123.1	2.2	2.1	2.2	15.5	14.9	16.1	65.3	62.3	68.3
2011	236.7	232.6	240.7	15.9	15.8	15.9	26.2	25.7	26.8	140.8	137.8	143.7
2012	299.5	295.5	303.6	15.3	15.2	15.3	6.8	6.3	7.4	239.4	236.4	242.4
2013	513.9	509.8	517.9	22.4	22.3	22.4	6.1	5.5	6.7	351.3	348.3	354.3
2014	584.8	580.7	588.9	34.8	34.8	34.9	15.2	14.6	15.8	396.8	393.8	399.8
2015	729.4	725.3	733.5	37.2	37.1	37.3	31.3	30.7	31.9	372.1	369.1	375.2
Constant	1,303.9	1,300.6	1,307.3	101.9	101.9	102.0	169.3	168.8	169.8	707.2	704.7	709.7

*Death: 1 if the individual died in the given year; 0 if the person did not die in the given year

**b: coefficient

Table 4 shows the impact of the number of chronic conditions on total healthcare costs. One chronic condition increased the annual costs with € 600 whereas five or more conditions increased the costs with € 3,949. Proportionally similar increases were seen for the categories of costs.

**Table 4:** Associations between the number of chronic conditions and healthcare costs (in Euro).

Cost types	Total cost			GP cost			Pharmacy cost			Hospital care cost		
	b**	95% CI		b**	95% CI		b**	95% CI		b**	95% CI	
Number of chronic conditions (reference: no chronic condition)												
1	600.6	595.3	606.0	21.5	21.4	21.5	137.2	136.4	137.9	311.5	307.6	315.5
2	1,202.5	1,195.0	1,210.0	39.3	39.2	39.5	291.7	290.6	292.8	559.3	553.8	564.8
3	1,869.9	1,860.4	1,879.3	58.7	58.6	58.9	470.1	468.7	471.5	792.9	785.9	799.9
4	2,701.0	2,689.1	2,713.0	80.5	80.3	80.7	675.9	674.2	677.7	1,110.7	1,101.9	1,119.6
5 or more	3,949.3	3,934.3	3,964.4	114.2	114.0	114.4	987.2	985.1	989.4	1,558.4	1,547.2	1,569.5
Death*	3,656.8	3,642.1	3,671.5	198.3	198.0	198.5	-273.9	-276.0	-271.7	2,987.7	2,976.8	2,998.5
Year (reference: 2009)												
2010	108.4	104.4	112.4	1.9	1.8	2.0	12.8	12.2	13.4	61.4	58.5	64.4
2011	212.5	208.4	216.5	15.2	15.1	15.3	20.1	19.5	20.7	131.9	129.0	134.9
2012	268.0	264.0	272.1	14.4	14.4	14.5	-1.2	-1.8	-0.6	227.9	224.9	230.9
2013	478.8	474.7	482.9	21.4	21.3	21.5	-2.9	-3.4	-2.3	338.6	335.6	341.6
2014	541.3	537.2	545.3	33.7	33.6	33.7	4.1	3.5	4.7	381.0	377.9	384.0
2015	676.4	672.3	680.5	35.7	35.7	35.8	17.8	17.2	1.8	352.9	349.8	355.9
Constant	1,244.6	1,241.2	1,247.9	100.3	100.2	100.3	154.1	153.6	154.6	685.6	683.2	688.1

*Death: 1 if the person died in that given year; 0 if the person did not die in the given year

**b: coefficient

## Discussion

Our results demonstrated that multimorbidity has not only become more prevalent (0.4 million additional people with multimorbidity) but also more severe in the Netherlands over the past ten years. The percentage of people with three or more conditions increased and this increase was highest in the category with five or more chronic conditions (0.8%-point). Hence, the people with multimorbidity have more chronic conditions with increases their complexity and further increases the challenges to the provision of integrated chronic care.

Compared with the latest reported Dutch study, our study showed a higher prevalence of multimorbidity but a slower rate of increase over time. This previous study reported a prevalence estimate of 16.2% in 2011 [17], while our estimate was 24.4% in the same year. Prevalence estimates may vary as a result of a difference in population studied, list of chronic conditions used, and source of data [56]. In contrast to our study, which was based on pharmacy data, this previous study was based on regional GP-registrations. It reported an increase in prevalence of multimorbidity of 0.4%-point per year, whereas our estimate showed 0.15%-point increase per year. Our estimate might be lower because we estimated the increase on the general population instead of regional GP-registrations.

Our study further showed that multimorbidity was associated with impaired physical and mental health. More importantly, for each additional chronic condition the additional impairment increased. The negative association between multimorbidity and physical health is in line with previous studies [20, 57, 58], among which two studies based on Dutch data [6, 7]. However, these Dutch studies included people of 85 years or over who already have a worse ADL score due to ageing alone. The negative association between multimorbidity and mental health is also in line with previous studies [23, 24], although Fortin et al. reported a weaker association [23]. The increasing impairment from each additional chronic disease further stresses the need to adopt a person-centred holistic approach instead of a disease-specific approach towards chronic disease management.

Our study clearly showed that multimorbidity disproportionally affected people with a lower education level. The negative impact of multimorbidity on ADL was about three times greater in those with the lowest education level than in those with the highest education level. The negative impact of mental health was about two times greater, respectively. We found no previous studies comparing the impact of multimorbidity between groups with different education levels. This result highlights the need to specifically engage the lower educated patients in chronic disease management and provide targeted self-management support to the lower educated patients.

Multimorbidity increased the total healthcare cost by € 874 as compared to people with a single chronic condition. This was largely due to an increase in costs of medication (+€ 355) and costs of hospital care (+€ 226). There is no evidence of diminishing increases as the number of conditions increases. The association between multimorbidity and costs was previously reported for Switzerland, Ireland, and the U.S. [32], and although they all found considerable increases, the absolute increases are difficult to compare because of difference in healthcare and insurance systems, prices, medical practice patterns and databases used. Previous Dutch studies [59, 60] have only reported the association between multimorbidity and healthcare utilization, but not costs.

### Strength and limitations

A strength of our study was the use of comprehensive databases, yielding representative results for the Netherlands. The Health Survey that was used to measure physical and mental health, is send to a random sample of the Dutch adult population, and bias due to non-response is reduced by weighting the data with respect to sex, age, ethnicity, marital status, geographic characteristics, and survey season [61]. The pharmacy database that we used to estimate the prevalence of multimorbidity covered over 90% of the population in the Netherlands. This pharmacy database includes all medication prescribed by GP’s, to hospital outpatients and patients in residential homes, but not medication prescribed during a hospital admission and the over-the-counter medication. However, it is unlikely that this limitation has affected the prevalence of multimorbidity because most chronic conditions require continuous pharmacotherapy with medicines that are only available on prescription from doctors and these prescriptions are generally filled at local outpatient pharmacies. The health insurance database we used included 98% of all declarations for services included in the basic benefit package. However, services covered by private health insurance and out-of-pocket expenditures were not included. In 2015, they amounted to about 4.4% and 14.2% of total healthcare costs, respectively [62]. Out-of-pocket payments mostly included the deductible of the basic health insurance (max €375 per person in 2015) and certain services like eyeglasses, contact lenses, inlay soles and certain dental prostheses that not covered by basic health insurance. The latter is generally not caused by or unique to certain chronic diseases. The deductible (the amount paid out of pocket by the patient before a health insurer will pay any expenses) can be substantial for patients with chronic diseases, but was not included because we only had data on costs covered by the basic health insurance.

While many chronic conditions can be identified well based on pharmacy data, some are more difficult. For example, a condition like chronic pain is challenging to identify since pain-controlling medications are also accessible as over-the-counter medication, which is not recorded in the pharmacy database. However, the stronger pain-controlling medications, such as morphine, fentanyl and oxycodone, are only accessible with specialists’ prescription, which are included on the pharmacy data. Additionally, the possibility of overestimating the prevalence of multimorbidity was reduced by applying the two-year continuously prescription criteria in defining chronic conditions. However, the prescription of preventive medication at the sub-clinical stage could lead to an overestimation. This especially pertains to hypertension-control. To investigate the potential influence on the results, a sensitivity analysis that excluded the first-line medications for hypertension-control (i.e. ATC code of diuretic agents) was performed. This analysis shows a similar result. The prevalence of multimorbidity is 1.5 to 2%-point lower than the original results and has a similar incremental trend.

A limitation of our study is that the Health Survey database only includes cross-sectional data [42]. Consequently, the analysis of these data precluded conclusions on the causal association between multimorbidity and physical and mental health outcomes [63]. On the other hand, the impact of multimorbidity on the healthcare cost was estimated in panel data, which does allow conclusions about causality since the unmeasured confounding factors and reversed causality can be better addressed with panel data [64].

The possible non-response bias is inevitable for most questionnaire-based studies, and there is no exception for the Health Survey database used in this study. However, this bias is addressed by a weighting that CBS applies to ensure representation by age, gender, marital status, province, household size, income and urbanity [61].

A further limitation is that we had to use ADL score was a proxy for physical health. The questions in the ADL questionnaire are more applicable to people who are more severely ill, which limits the assessment of subtle influences of chronic conditions on physical functioning [65]. In future surveys a more comprehensive measure of physical health should be used.

Additionally, for future studies it would be interesting to analyse disease clusters to have more insight in the effect of certain diseases combinations. That would, however, require a larger sample size than in our current study.

### Implications

The implications of these findings are that they raise awareness of the increasing problem of multimorbidity, which calls for greater efforts in trying to limit the impact thereof on persons’ lives. These efforts should specifically target the more vulnerable groups such as those with a lower education level. Person-centred integrated health and social care initiatives are expected to contribute to the solution. However, surprisingly few of them offer additional or specific services and support for lower educated groups. While recognizing it won’t be easy to reach these groups, there is definitely a window of opportunity there. However, when more attention is being paid to integrated care for people with a lower education level, the risk of stigmatizing this group increases. Although there is an association between lower education level and worse health, the majority of the population, also from the lower educated groups, is healthy. This calls for carefully targeting interventions to people within the lower educated group that need support most.

## Conclusion

This analysis of nationally representative data from the Netherlands has shown that multimorbidity is associated with seriously impaired physical and mental health and disproportionally affects those with a lower education level. The impact of each additional chronic condition seems to grow as the number of chronic conditions increases. Each additional chronic condition leads to a considerable increase in costs, especially for medication and hospital costs, and there is no evidence of diminishing impact as the number of chronic conditions increases.

## Supporting information

S1 Table Prevalence of chronic diseases and multimorbidity from 2007 to 2016. (DOCX)

S2 Table Associations between multimorbidity and health outcomes across education levels. (DOCX)

S3 Table The demographic summary for the Health Survey database. (DOCX)

S1 Appendix Stata code for statistical analyses. (DOCX)
