# Supplementary material for: The disease burden of multimorbidity and its interaction with educational level
Source: PLoS One. 2020 Dec 3;15(12):e0243275. doi: 10.1371/journal.pone.0243275 (PMC7714131; doi:10.1371/journal.pone.0243275)
Supplement: S1 Table — (DOCX) [file pone.0243275.s001.docx]

S1 Table. Prevalence of chronic diseases and multimorbidity from 2007 to 2016

| **Multimorbidity in men and women (%)** | | | | | | | | | | |
| --- | --- | --- | --- | --- | --- | --- | --- | --- | --- | --- |
|  | **2007** | **2008** | **2009** | **2010** | **2011** | **2012** | **2013** | **2014** | **2015** | **2016** |
| **Overall** | 23.2 | 23.9 | 23.0 | 23.6 | 24.4 | 24.3 | 24.5 | 24.3 | 24.5 | 24.7 |
| **18-29** | 2.5 | 2.6 | 2.3 | 2.4 | 2.6 | 2.3 | 2.2 | 2.1 | 2.2 | 2.2 |
| **30-39** | 6.7 | 6.9 | 6.2 | 6.3 | 6.6 | 6.0 | 5.7 | 5.6 | 5.6 | 5.6 |
| **40-49** | 14.3 | 14.7 | 13.5 | 13.7 | 14.0 | 13.3 | 12.5 | 12.4 | 12.3 | 12.3 |
| **50-59** | 26.4 | 27.2 | 25.9 | 26.4 | 27.2 | 26.4 | 25.7 | 25.4 | 25.2 | 24.9 |
| **60-69** | 43.8 | 44.0 | 42.0 | 42.7 | 43.9 | 43.7 | 43.6 | 42.8 | 43.1 | 41.8 |
| **70-79** | 60.6 | 62.0 | 60.1 | 60.9 | 61.8 | 61.9 | 64.3 | 62.4 | 62.1 | 64.4 |
| **80+** | 71.7 | 73.5 | 71.1 | 72.5 | 73.7 | 74.4 | 76.5 | 75.0 | 75.6 | 76.2 |
| **Number of chronic conditions in men and women (%)** | | | | | | | | | | |
| **1** | 18.7 | 18.7 | 18.9 | 18.7 | 18.5 | 18.4 | 18.2 | 17.9 | 17.7 | 17.5 |
| **2** | 10.9 | 11.0 | 10.8 | 10.9 | 10.9 | 10.7 | 10.6 | 10.5 | 10.4 | 10.4 |
| **3** | 6.4 | 6.6 | 6.4 | 6.6 | 6.8 | 6.8 | 6.8 | 6.9 | 7.0 | 7.0 |
| **4** | 3.3 | 3.5 | 3.3 | 3.5 | 3.7 | 3.7 | 3.7 | 3.8 | 3.9 | 4.0 |
| **5+** | 2.6 | 2.9 | 2.5 | 2.7 | 3.0 | 3.1 | 3.0 | 3.1 | 3.2 | 3.4 |
| **Multimorbidity in men (%)** | | | | | | | | | | |
|  | **2007** | **2008** | **2009** | **2010** | **2011** | **2012** | **2013** | **2014** | **2015** | **2016** |
| **Overall** | 20.0 | 20.7 | 20.4 | 21.1 | 21.9 | 22.0 | 22.0 | 22.3 | 22.7 | 22.9 |
| **18-30** | 1.5 | 1.6 | 1.5 | 1.5 | 1.6 | 1.5 | 1.4 | 1.4 | 1.4 | 1.4 |
| **31-40** | 5.0 | 5.2 | 4.6 | 4.7 | 4.9 | 4.5 | 4.3 | 4.2 | 4.3 | 4.3 |
| **41-50** | 11.5 | 11.8 | 11.0 | 11.2 | 11.5 | 10.9 | 10.4 | 10.3 | 10.3 | 10.3 |
| **51-60** | 23.9 | 24.5 | 23.8 | 24.2 | 24.9 | 24.2 | 23.7 | 23.4 | 23.3 | 23.0 |
| **61-70** | 41.9 | 42.3 | 41.6 | 42.4 | 43.5 | 43.5 | 43.1 | 43.1 | 43.5 | 42.2 |
| **71-80** | 59.0 | 60.5 | 60.0 | 61.1 | 62.0 | 62.3 | 62.7 | 63.1 | 62.9 | 65.5 |
| **80+** | 70.5 | 72.8 | 72.3 | 74.4 | 76.1 | 77.0 | 77.0 | 78.2 | 79.0 | 79.7 |
| **Number of chronic conditions in men (%)** | | | | | | | | | | |
| 1 | 17.0 | 17.0 | 17.1 | 16.9 | 16.7 | 16.6 | 16.3 | 16.0 | 15.8 | 15.7 |
| 2 | 10.0 | 10.2 | 10.1 | 10.2 | 10.3 | 10.1 | 10.0 | 10.0 | 9.9 | 9.8 |
| 3 | 5.6 | 5.9 | 5.9 | 6.1 | 6.3 | 6.4 | 6.5 | 6.6 | 6.8 | 6.9 |
| 4 | 2.6 | 2.8 | 2.7 | 2.9 | 3.2 | 3.2 | 3.2 | 3.4 | 3.5 | 3.6 |
| 5+ | 1.7 | 1.9 | 1.8 | 1.9 | 2.2 | 2.3 | 2.2 | 2.3 | 2.5 | 2.6 |
| **Multimorbidity in women (%)** | | | | | | | | | | |
|  | **2007** | **2008** | **2009** | **2010** | **2011** | **2012** | **2013** | **2014** | **2015** | **2016** |
| **Overall** | 26.2 | 27.0 | 25.4 | 26.0 | 26.8 | 26.5 | 26.9 | 26.2 | 26.3 | 26.5 |
| **18-30** | 3.4 | 3.6 | 3.2 | 3.4 | 3.6 | 3.2 | 3.0 | 2.9 | 3.0 | 3.0 |
| **31-40** | 8.5 | 8.7 | 7.7 | 7.9 | 8.2 | 7.5 | 7.0 | 7.0 | 7.0 | 7.0 |
| **41-50** | 17.2 | 17.6 | 16.0 | 16.2 | 16.6 | 15.7 | 14.7 | 14.5 | 14.3 | 14.3 |
| **51-60** | 29.0 | 30.0 | 28.0 | 28.6 | 29.5 | 28.7 | 27.9 | 27.3 | 27.1 | 26.8 |
| **61-70** | 45.6 | 45.7 | 42.5 | 43.1 | 44.2 | 43.9 | 44.2 | 42.5 | 42.7 | 41.4 |
| **71-80** | 62.0 | 63.2 | 60.1 | 60.8 | 61.6 | 61.6 | 65.6 | 61.7 | 61.3 | 63.4 |
| **80+** | 72.3 | 73.8 | 70.5 | 71.5 | 72.5 | 73.0 | 76.3 | 73.2 | 73.7 | 74.2 |
| **Number of chronic conditions in women (%)** | | | | | | | | | | |
| **1** | 20.4 | 20.3 | 20.7 | 20.5 | 20.2 | 20.2 | 20.1 | 19.7 | 19.5 | 19.2 |
| **2** | 11.7 | 11.8 | 11.5 | 11.5 | 11.6 | 11.3 | 11.2 | 11.1 | 11.0 | 10.9 |
| **3** | 7.2 | 7.3 | 7.0 | 7.1 | 7.3 | 7.2 | 7.1 | 7.1 | 7.2 | 7.2 |
| **4** | 4.0 | 4.2 | 3.8 | 4.0 | 4.2 | 4.2 | 4.1 | 4.2 | 4.3 | 4.3 |
| **5+** | 3.4 | 3.8 | 3.2 | 3.4 | 3.7 | 3.8 | 3.7 | 3.8 | 3.9 | 4.1 |
