# Supplementary material for: The disease burden of multimorbidity and its interaction with educational level
Source: PLoS One. 2020 Dec 3;15(12):e0243275. doi: 10.1371/journal.pone.0243275 (PMC7714131; doi:10.1371/journal.pone.0243275)
Supplement: S2 Table — (DOCX) [file pone.0243275.s002.docx]

S2 Table. Associations between multimorbidity and health outcomes across education levels

| Health outcome | ADL | | | | | | MHI | | |
| --- | --- | --- | --- | --- | --- | --- | --- | --- | --- |
| Model | Logistic | | | GLM | | | OLS | | |
|  | OR^**^ | 95% CI | | Exp(b) | 95% CI | | b | 95% CI | |
| Age | 1.03 | 1.02 | 1.03 | 0.99 | 0.99 | 0.99 | 0.09 | 0.08 | 0.10 |
| Female | 1.40 | 1.23 | 1.58 | 1.16 | 1.06 | 1.28 | -1.65 | -2.00 | -1.29 |
| Education level^*^ (benchmark: 1) | | | | | | | | | |
| 2 | 0.72 | 0.39 | 1.33 | 0.55 | 0.31 | 0.97 | -0.09 | -1.11 | 0.93 |
| 3 | 0.59 | 0.33 | 1.03 | 0.73 | 0.43 | 1.24 | 1.08 | 0.18 | 1.98 |
| 4 | 0.56 | 0.29 | 1.07 | 0.49 | 0.27 | 0.89 | 0.94 | -0.04 | 1.91 |
| 5 | 0.61 | 0.28 | 1.31 | 0.40 | 0.20 | 0.81 | 0.98 | -0.14 | 2.11 |
| Morbidity condition (benchmark: no morbidity) | | | | | | | | | |
| Single morbidity | 2.89 | 1.56 | 5.37 | 1.06 | 0.61 | 1.82 | -2.57 | -4.04 | -1.10 |
| Multimorbidity | 13.52 | 8.38 | 21.81 | 1.26 | 0.83 | 1.91 | -6.41 | -7.49 | -5.33 |
| Morbidity condition # Education level | | | | | | | | | |
| Single #Edu. 2 | 1.36 | 0.60 | 3.06 | 1.30 | 0.63 | 2.67 | 0.57 | -1.28 | 2.41 |
| Single #Edu. 3 | 1.18 | 0.55 | 2.51 | 0.90 | 0.46 | 1.77 | 0.95 | -0.69 | 2.60 |
| Single #Edu. 4 | 1.36 | 0.58 | 3.18 | 1.24 | 0.58 | 2.64 | 1.42 | -0.37 | 3.21 |
| Single #Edu. 5 | 0.85 | 0.31 | 2.33 | 2.11 | 0.85 | 5.24 | 0.79 | -1.19 | 2.78 |
| Multi # Edu. 2 | 1.01 | 0.52 | 1.96 | 1.46 | 0.81 | 2.62 | 1.39 | 0.02 | 2.76 |
| Multi # Edu. 3 | 1.08 | 0.59 | 2.00 | 0.92 | 0.53 | 1.58 | 1.06 | -0.19 | 2.30 |
| Multi # Edu. 4 | 0.97 | 0.48 | 1.96 | 1.27 | 0.68 | 2.38 | 2.65 | 1.23 | 4.08 |
| Multi # Edu. 5 | 0.54 | 0.23 | 1.26 | 1.78 | 0.83 | 3.82 | 2.90 | 1.16 | 4.64 |
| _cons | 0.05 | 0.03 | 0.08 | 1.20 | 0.81 | 1.78 | 5.94 | 5.87 | 6.02 |

*Education level: 1 for primary school; 2 for Pre-vocational training; 3 for High school or vocational training; 4 for Higher education until Bachelor; 5 for Master/doctorate.

**OR: odds ratio
