# Supplementary material for: The disease burden of multimorbidity and its interaction with educational level
Source: PLoS One. 2020 Dec 3;15(12):e0243275. doi: 10.1371/journal.pone.0243275 (PMC7714131; doi:10.1371/journal.pone.0243275)
Supplement: S3 Table — (DOCX) [file pone.0243275.s003.docx]

S3 Table. The Demographic Summary for Health Survey database (N=7,741)

| Average age (year) | 52.8 |
| --- | --- |
| Gender (male) | 50.5% |
| Education level | |
| Level 1 | 12.8% |
| Level 2 | 21.0% |
| Level 3 | 38.3% |
| Level 4 | 18.3% |
| Level 5 | 9.6% |
