# Supplementary material for: The disease burden of multimorbidity and its interaction with educational level
Source: PLoS One. 2020 Dec 3;15(12):e0243275. doi: 10.1371/journal.pone.0243275 (PMC7714131; doi:10.1371/journal.pone.0243275)
Supplement: S1 Appendix — (DOCX) [file pone.0243275.s004.docx]

S1 Appendix. Stata code for statistical analyses

Physical health outcome analysis_3lv:

Part 1:

Logistic ADL01 c.age i.gender i.edulv i.MM_3lv

Part 2:

Glm ADL c.age i.gender i.edulv i.MM_3lv, link(log) family(gamma), if ADL01==1

Physical health outcome analysis_6lv:

Part 1:

Logistic ADL01 c.age i.gender i.edulv i.MM_6lv

Part 2:

Glm ADL c.age i.gender i.edulv i.MM_6lv, link(log) family(gamma), if ADL01==1

Physical health outcome analysis_3lv with interaction:

Part 1:

Logistic ADL01 c.age i.gender i.edulv i.MM_3lv i.MM_3lv# i.edulv

Part 2:

Glm ADL c.age i.gender i.edulv i.MM_3lv i.MM_3lv# i.edulv, link(log) family(gamma), if ADL01==1

Mental health outcome analysis_3lv:

Reg MHI c.age i.gender i.edulv i.MM_3lv

Mental health outcome analysis_6lv:

Reg MHI c.age i.gender i.edulv i.MM_6lv

Mental health outcome analysis_3lv with interaction:

Reg MHI c.age i.gender i.edulv i.MM_3lv i.MM_3lv# i.edulv

Healthcare cost analysis_3lv:

Total healthcare cost: Xtreg cost_t i.MM_3lv i.die i.year, fe

Hospital care cost: Xtreg cost_h i.MM_3lv i.die i.year, fe

Pharmacy care cost: Xtreg cost_p i.MM_3lv i.die i.year, fe

GP care cost: Xtreg cost_g i.MM_3lv i.die i.year, fe

Healthcare cost analysis_6lv:

Total healthcare cost: Xtreg cost_t i.MM_6lv i.die i.year, fe

Hospital care cost: Xtreg cost_h i.MM_6lv i.die i.year, fe

Pharmacy care cost: Xtreg cost_p i.MM_6lv i.die i.year, fe

GP care cost: Xtreg cost_g i.MM_6lv i.die i.year, fe
